# Survival trends in hematological malignancies in the Nordic countries through 50 years

**DOI:** 10.1038/s41408-022-00728-z

**Published:** 2022-11-07

**Authors:** Kari Hemminki, Janne Hemminki, Asta Försti, Amit Sud

**Affiliations:** 1 Biomedical Center, Faculty of Medicine in Pilsen, Charles University in Prague, Pilsen, Czech Republic; 2 Division of Cancer Epidemiology, German Cancer Research Center (DKFZ), Heidelberg, Germany; 3 Hopp Children’s Cancer Center (KiTZ), Heidelberg, Germany; 4 Division of Pediatric Neurooncology, German Cancer Research Center (DKFZ), German Cancer Consortium (DKTK), Heidelberg, Germany; 5 Division of Genetics and Epidemiology, The Institute of Cancer Research, London, UK; 6 Haemato-oncology Unit, The Royal Marsden Hospital NHS Foundation Trust, Sutton, UK

**Keywords:** Epidemiology, Prognosis

## Abstract

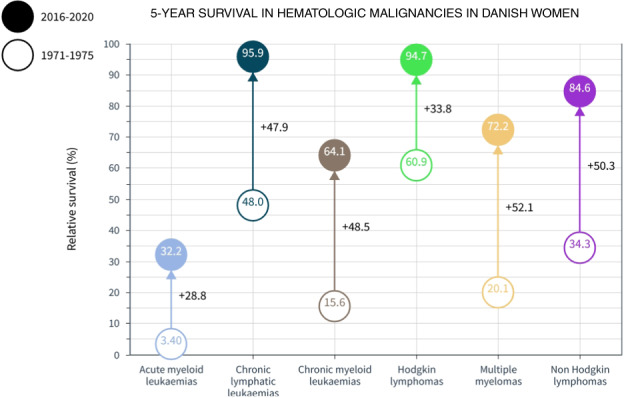

Improving cancer survival without compromising the quality of life is the main challenge for medical oncology. Since the introduction of traditional chemotherapies from the 1950s, a number of diverse treatment modalities have been employed to improve the survival of individuals with hematological malignancies (HMs) [1]. Two HMs, Hodgkin lymphoma (HL) and childhood acute lymphatic leukemia (ALL) were among the first cancers for which high cure rates were achieved and serve as success stories in oncology. In HL, risk-adapted therapies were introduced with intensive poly-chemotherapeutic regimens in combination with other modalities [2]. In childhood ALL, treatment was based on risk stratification, assessment of minimal residual disease and additional supportive care [3]. Therapies for these HMs were optimized in clinical randomized trials and are increasingly being administered in specialized centers.

Recent survival studies on HMs from Europe and USA have shown a generally positive trend. However, most studies have covered a short follow-up period because of diagnostic uncertainties and a lack of data [4, 5]. The Nordic cancer registries are the oldest national cancer registries in the world and cover almost all cancers with complete follow-up [6]. Grouped data from these cancer registries are publicly available in the NORDCAN database, which has been used in survival studies in HMs starting from the 1960s [7]. Here, we use this resource, recently extended to include data to the year 2020, in an analysis of survival in all available specific HMs from Denmark (DK), Finland (FI), Norway (NO) and Sweden (SE). We show data on 1-year and 5-year relative survival through a half century.

The NORDCAN database was accessed at the IARC website (https://nordcan.iarc.fr/en/database#bloc2). The current survival data cover 5-year periods from 1971 through 2020. The survival methods are described at the NORDCAN website and elsewhere [8]. The analysis included HMs, as listed in Supplementary Table 1 with the used International Classification of Diseases (ICD) version 10 codes and case numbers. Data for unspecified HMs were not considered. Data for ALL were not included as it was not possible to distinguish childhood and adult ALL. Less than 50 years of data were available on myelodysplastic syndrome (MDS), in DK and SE since 1980 and in FI and NO later; on myeloproliferative diseases (MPD), data were available in DK since 1980 and in NO later. Age-group specific survival analysis was not available in the latest update of NORDCAN. Instead this was done on a previous version and covered the latest available 5-year period, 2012–16. We considered survival for all patients and in the oldest age group (70–89 years).

Relative 1- and 5-year survival for men and women of each Nordic county is shown in Fig. 1 (note, data from 1970 to 2019). For HMs associated with a high relative survival, such as non-Hodgkin lymphoma (NHL), HL and chronic lymphocytic leukemia (CLL), 1-year and 5-year survival points in the different countries cluster together, while for multiple myeloma (MM), MDS and acute myeloid leukemia (AML), relative survival points separated. Female 5-year survival was better than male survival for NHL, MDS, MPD and CML. Data points for DK and SE are often on top, showing best survival, and those for FI are lowest in almost all HMs.

**Fig. 1:**
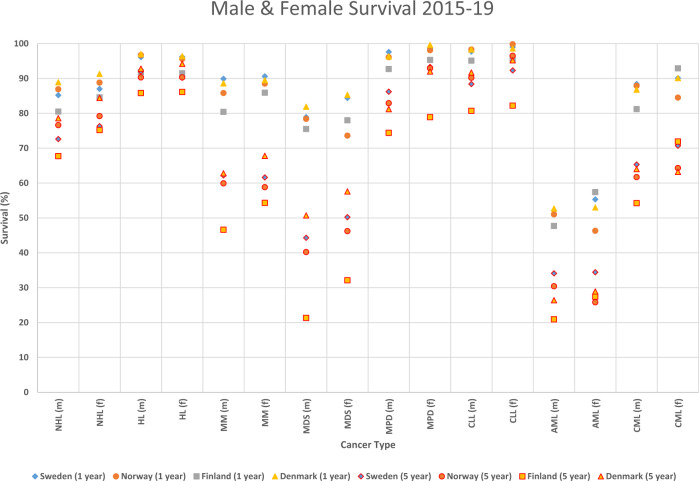
Survival in hematological malignancies in the Nordic countries in 2015–19. Relative 1- and 5-year survival in men and women is shown separately for each country according to the Nordcan database. Data points for 1-year survival cluster on top and for 5-year survival in bottom.

To assess the change in survival from 1971–75 to 2016–20, we collated data for the first and the last 5-year period for SE men and women (Fig. 2A, B). Among men, the largest improvements were observed for CML (46.8 % units), CLL (45.8 % units) and NHL (41.1 % units). The smallest improvement of 22.8% units was for MPD. Among women, the major difference from men was the very large increase in survival in CML (57.6 % units).

**Fig. 2:**
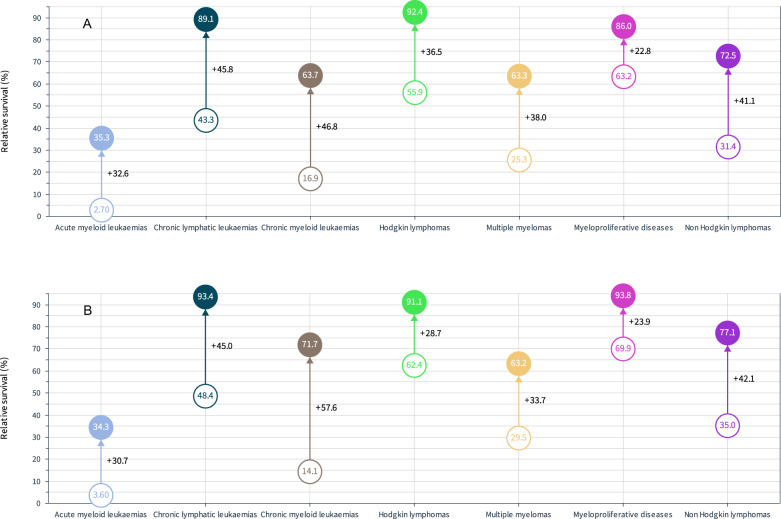
Improvement in relative 5-year survival in hematological malignancies between 1971–75 and 2016–20 in Sweden according to the Nordcan database. Data for Swedish men (**A**) and women (**B**). The figures inside the circles show survival % in the first and last 5-year period. The figure son the side of each arrow show the improvement in absolute % units.

Improvements in 5-year survival for many HMs were most favorable in DK among the Nordic countries (Fig. 1). In Supplementary Fig. 1 male data are shown from 1971–1975 to 2016–2020. Improvement in CLL, CML, MM and NHL were more than 50% units reaching 93.2%, 66.8%, 66.5%, and 79.6% units. For women the development was equally impressive and many of the survival figures, such as 72.2% for MM may be an international record (Supplementary Fig. 2).

To assess periodic development in survival, we plotted 5-year survival increase for SE men between years 1996–2000 and 2016–2020, and we marked the starting level in period 1971–1975 with ‘x’ (Supplementary Fig. 3). For HMs of good survival (HL and MPD) the recent improvement has been modest compared to the early period but for MM (25.9% units) and CML (22.6% units) recent development has been favorable. For AML, recent development accounted for most of the survival gains. For women, recent gain of 31.6% units for CML has been particularly favorable, explaining the female survival advantage at the end (71.7% vs 63.7 in men) (Supplementary Fig. 4).

Age-group specific 5-survival data in 2012–2016 are shown in Supplementary Table 2 for all patients and for those in the oldest age group (70–89 years). Survival in age group 70 to 89 was consistently lower than that for all patients with a difference in CLL of approximately 20%, and for HL, MM, MDS, MPD and CML a difference of approximately 50%, but there was variation between the countries. For AML, survival in the 70–89 year age group was strikingly low at 1 or 2% in all countries. In those years, older patients (> 70 years of age) accounted for half of all HMs (48% of male and 52% of female HMs).

Survival has improved for the HMs over the past 50 years in the Nordic countries, ranging from 20 to more than 50% of units. A number of factors are likely to explain such progress and include centralization of care, earlier diagnoses, enhanced risk stratification including more sensitive disease detection, the use of novel therapies, optimization of existing treatments and better supportive care. We are aware of the recent introduction of numerous novel therapies in hematology practice in the form of immunotherapy and small molecule inhibitors which may not be captured in the 5-year survival in the last time period (2016–2020) in NORDCAN because the survival method generates the data for the last 5-year period by comparing to the previous period [9].

While 1-year survival was better than 80% for all HMs in all or most Nordic countries (2015–2019), with the exception of AML, 5-year survival showed distinct differences between the types of HMs. Survival was over 90% for HL, MPD and CLL, 60% for MM and CML, 50% for MDS and 30% for AML. The female 5-year survival was better than male survival in NHL, MDS, MPD and CML. Most encouragingly, 5-year survival increased markedly for CML, MM and AML for which few patients survived 5 years in the 1970s. However, the age-related disadvantage was marked in all Nordic countries, as elsewhere [10]. The situation was embracing for AML, for which no more than 1 or 2% of the 70–89 year old patients were alive in 5 years. For CLL the age-related survival difference was small but for the other HMs it was approximately 50%.

The therapeutic landscape of the Nordic countries with their centrally organized health care differs extensively from the heterogeneous US system but the survival outcomes are not very different, when the present data are compared with 5-year survival data from the Surveillance, Epidemiology and End Results (SEER) program for years 2012–18 (https://seer.cancer.gov/statfacts/). For MM, CLL and particularly for MDS, SE/DK 5-year survival is more than 5 % units over the SEER results. The SEER survival for CML is better than the SE/DK data.

The NORDCAN data are limited in specifying the exact type of HM, which for NHL and MPD implies a combination of vastly different subtypes [4, 5]. Nevertheless, for each of HM there are subtypes with diverse risk profiles and with survival implications. Stage and treatment information is lacking and thus adjustment or stratification in survival data are not possible. To compensate for these deficits, NORDCAN is the only database that offers high-quality nation-wide cancer data over a half century and thus enable analysis of long-term survival improvements. HL has remained an example for HM for which the therapeutic armamentarium has remained largely constant but its optimized use has enabled high cure rates [11]. Also survival in AML and CLL have improved while existing therapies were optimized [12, 13]. However, novel therapies in HMs have transformed the management of MM and CML with coincident improvement in relative survival [14, 15].

In conclusion, there has been a general improvement in survival in patients diagnosed with HMs over the past 50 years. 5-year survival has reached greater than 90% in HL, MPD and CLL, and more than 50% for all others, but MDS and AML. For all HMs, 5-year survival increased between 20 and 50% units over the period. Even for the HMs which are still below the 90% mark, survival developed favorably. Survival in old patients remains a challenge but the reward would be a great boost to the overall survival, as the age group 70+ accounts for 50% of all HM patients.

## Supplementary information

Combined supplementary material

## Data Availability

Publically available data were used from the NORDCAN database.
