# Supplementary material for: Survival trends in hematological malignancies in the Nordic countries through 50 years
Source: Blood Cancer J. 2022 Nov 7;12(11):150. doi: 10.1038/s41408-022-00728-z (PMC9637692; doi:10.1038/s41408-022-00728-z)

SURVIVAL TRENDS IN HEMATOLOGICAL MALIGNANCIES IN THE NORDIC COUNTRIES THROUGH 50 YEARS

Kari Hemminki[1,2], Janne Hemminki[1], Asta Försti[3,4] Amit Sud[5,6]

[1] Biomedical Center, Faculty of Medicine and Biomedical Center in Pilsen, Charles University in Prague, 30605 Pilsen, Czech Republic;

[2] Division of Cancer Epidemiology, German Cancer Research Center (DKFZ), Im Neuenheimer Feld 580, D-69120, Heidelberg, Germany;

[3] Hopp Children's Cancer Center (KiTZ), Heidelberg, Germany;

[4]Division of Pediatric Neurooncology, German Cancer Research Center (DKFZ), German Cancer Consortium (DKTK), Heidelberg, Germany;

[5]Division of Genetics and Epidemiology, The Institute of Cancer Research, London, UK

[6]Haemato-oncology Unit, The Royal Marsden Hospital NHS Foundation Trust, Sutton, UK

Correspondence: Kari Hemminki, Biomedical Center, Faculty of Medicine and Biomedical Center in Pilsen, Charles University in Prague, 30605 Pilsen, Czech Republic.

Telephone: +496221421800
Fax: +496221422203

Email: K.Hemminki@dkfz.de

Running title: Survival improvements.

Key words: prognosis, periodic survival, treatment, cancer registry.

Supplementary Table 1. Numbers of patients diagnosed with the defined hematological neoplasms in years 2015-19

**Neoplasm**	**ICD-10 codes**	**Males**				**Females**			
		Denmark	Finland	Norway	Sweden	Denmark	Finland	Norway	Sweden
Non Hodgkin lymphoma	C82-86	4120	3458	2732	5016	3116	2985	2088	3694
Hodgkin lymphoma	C81	456	489	455	625	333	363	314	472
Multiple myeloma	C90	1559	1014	1394	2289	1123	940	1024	1638
Myelodysplastic syndrome	D46	969	379	553	1002	647	307	356	677
Myeloproliferative disease	D45+D47.1,3-5	1540	836	880	1424	1743	981	962	1554
Acute lymphatic leukaemia	C91.0	203	228	225	362	137	170	163	289
Chronic lymphatic leukaemia	C91.1	1533	978	1101	1946	935	550	700	1140
Acute myeloid leukaemia	C92.0+C93.0+C94.0+C94.2+C94.4-5	512	449	453	840	437	416	405	768
Chronic myeloid leukaemia	C92.1+C93.1+C94.1	404	173	287	476	265	143	179	375

Supplementary Table 2. Relative 5-year survival (%) for all patients and for the aged (diagnosed at age 70-89 years), 2012-2016.

Men	Denmark		Finland		Norway		Sweden	
	**All**	**Old**	**All**	**Old**	**All**	**Old**	**All**	**Old**
Acute myeloid leukemia	22	2	19	1	19	1	27	1
Chronic lymphatic leukemia	84	64	77	44	85	60	81	53
Chronic myeloid leukemia	56	35	58	23	53	22	64	35
Hodgkin lymphoma	89	66	86	39	84	28	86	43
Multiple myeloma	55	25	43	16	49	24	54	20
Myelodysplastic syndromes	42	25	15	1	37	14	42	24
Myeloproliferative diseases	75	46	69	36	80	45	-	-

Women	Denmark		Finland		Norway		Sweden	
	**All**	**Old**	**All**	**Old**	**All**	**Old**	**All**	**Old**
Acute myeloid leukemia	22	-	26	2	20	-	25	2
Chronic lymphatic leukemia	91	81	82	55	92	70	88	73
Chronic myeloid leukemia	67	20	63	26	61	12	66	38
Hodgkin lymphoma	87	48	89	58	88	45	89	59
Multiple myeloma	58	37	46	17	51	23	53	20
Myelodysplastic syndromes	51	31	24	9	43	25	47	35
Myeloproliferative diseases	88	70	78	55	91	73	-	-

Supplementary Fig. 1. Improvement in relative 5-year survival in hematological malignancies between 1971-1975 and 2016-2020 in Danish men. The figures inside the circles show survival % in the first and last 5-year period. The figures on the side of each arrow show the improvement in % units. No data are shown for myelodysplastic syndrome because missing periods.

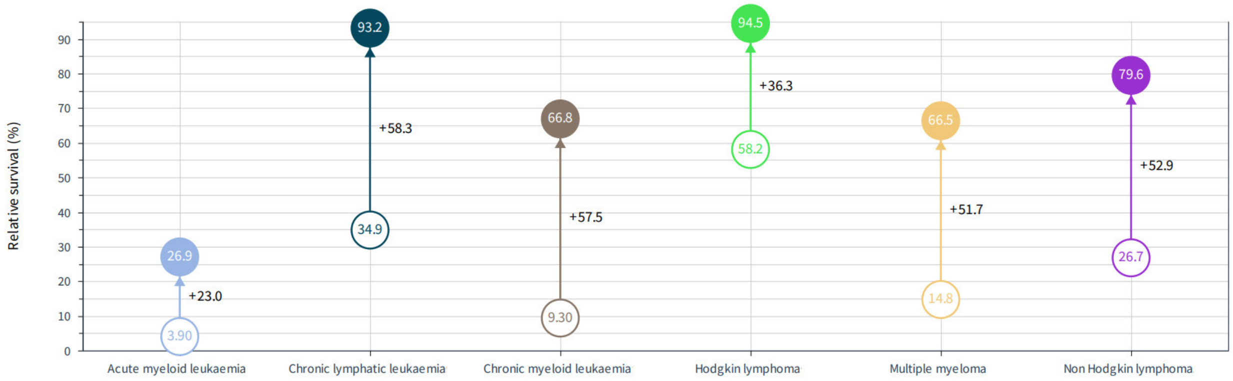

Supplementary Fig. 2. Improvement in relative 5-year survival in hematological malignancies between 1971-1975 and 2016-2020 in Danish women. The figures inside the circles show survival % in the first and last 5-year period. The figures on the side of each arrow show the improvement in % units. No data are shown for myelodysplastic syndrome because missing periods.

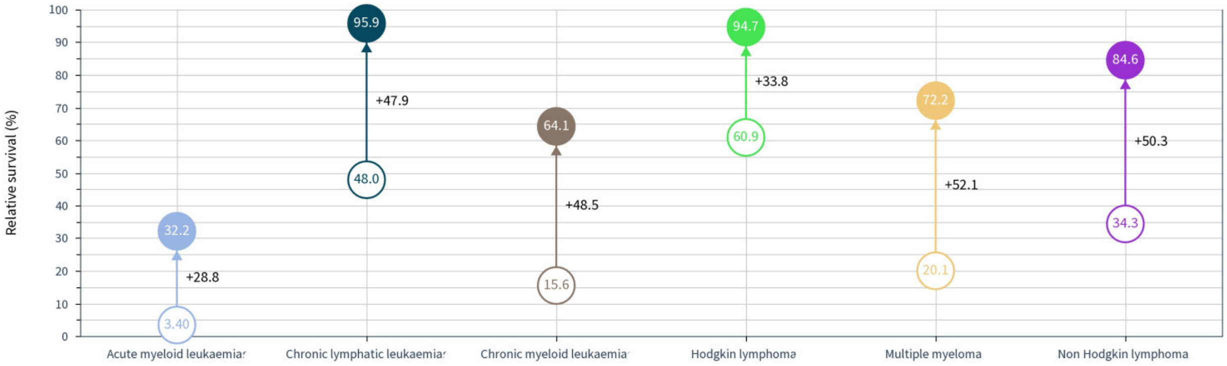

Supplementary Fig. 3. Improvement in relative 5-year survival in hematological malignancies between 1996-2000 and 2016-2020 in Swedish men. The figures inside the circles show survival % in the first and last 5-year period. The figures on the side of each arrow show the improvement in % units. The cross (x) below the lower circle gives the survival (% units) in period 1971-1975.

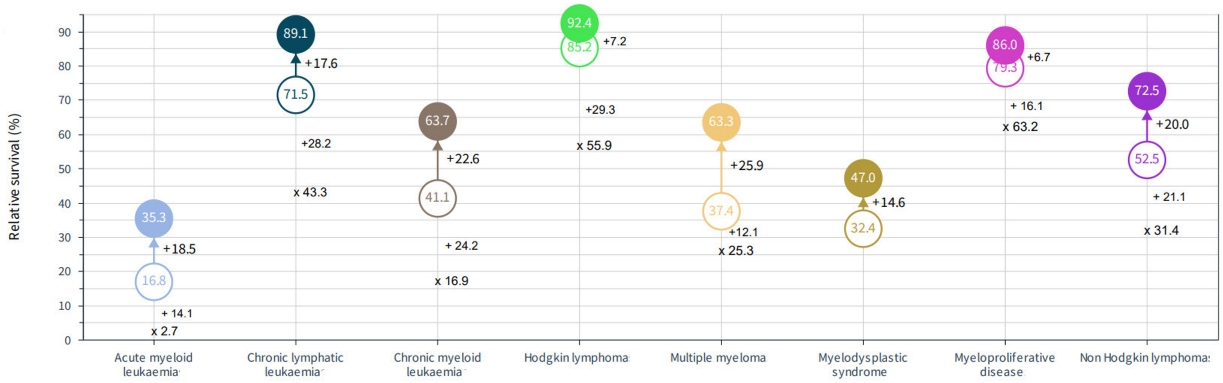

Supplementary Fig. 4. Improvement in relative 5-year survival in hematological malignancies between 1996-2000 and 2016-2020 in Swedish women. The figures inside the circles show survival % in the first and last 5-year period. The figures on the side of each arrow show the improvement in % units. The cross (x) below the lower circle gives the survival (% units) in period 1971-1975.

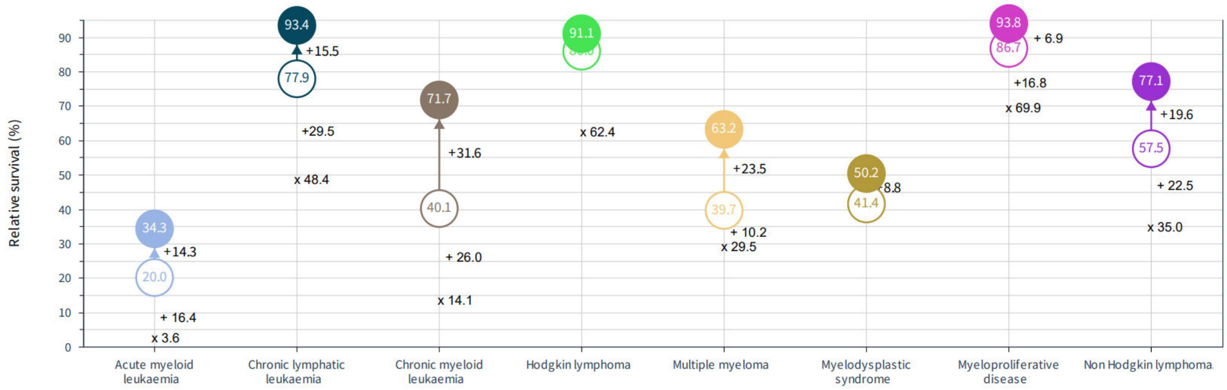

Supplement: Supplementary file 1 — Combined supplementary material [file 41408_2022_728_MOESM1_ESM.pdf]
